# Video-Based Communication Assessment: Development of an Innovative System for Assessing Clinician-Patient Communication

**DOI:** 10.2196/10400

**Published:** 2019-02-14

**Authors:** Kathleen M Mazor, Ann M King, Ruth B Hoppe, Annie O Kochersberger, Jie Yan, Jesse D Reim

**Affiliations:** 1 Meyers Primary Care Institute University of Massachusetts Medical School, Reliant Medical Group and Fallon Health Plan Worcester, MA United States; 2 National Board of Medical Examiners Philadelphia, PA United States; 3 College of Human Medicine Michigan State University East Lansing, MI United States

**Keywords:** communication, crowdsourcing, health care, mobile phone, patient-centered care, video-based communication assessment

## Abstract

Good clinician-patient communication is essential to provide quality health care and is key to patient-centered care. However, individuals and organizations seeking to improve in this area face significant challenges. A major barrier is the absence of an efficient system for assessing clinicians’ communication skills and providing meaningful, individual-level feedback. The purpose of this paper is to describe the design and creation of the Video-Based Communication Assessment (VCA), an innovative, flexible system for assessing and ultimately enhancing clinicians’ communication skills. We began by developing the VCA concept. Specifically, we determined that it should be convenient and efficient, accessible via computer, tablet, or smartphone; be case based, using video patient vignettes to which users respond as if speaking to the patient in the vignette; be flexible, allowing content to be tailored to the purpose of the assessment; allow incorporation of the patient’s voice by crowdsourcing ratings from analog patients; provide robust feedback including ratings, links to highly rated responses as examples, and learning points; and ultimately, have strong psychometric properties. We collected feedback on the concept and then proceeded to create the system. We identified several important research questions, which will be answered in subsequent studies. The VCA is a flexible, innovative system for assessing clinician-patient communication. It enables efficient sampling of clinicians’ communication skills, supports crowdsourced ratings of these spoken samples using analog patients, and offers multifaceted feedback reports.

## Introduction

Good clinician-patient communication is essential to quality health care and a key element of patient-centered care [1,2]. There is a substantial growing evidence base documenting the critical importance of effective clinician-patient communication for a variety of health outcomes [3,4]. Training in effectively communicating with patients and families is required for medical school and residency program accreditation [5,6], and competency in communication is a requirement for licensure [2,7]. Financial incentives for excellent clinician-patient communication and penalties for poor communication are becoming widespread [8-10]. Many practicing physicians are finding that a portion of their compensation is dependent on their patients’ perceptions of their communication skills, and health care reimbursement rates are increasingly influenced by patients’ ratings of communication [11-13]. In short, research evidence as well as societal and financial factors and policies have converged to influence health care systems and individual clinicians to value communication and seek to improve in this area.

Although the importance of high-quality clinician-patient communication is widely acknowledged, individuals and organizations seeking to improve in this area face significant challenges. Medical schools and some residencies utilize standardized patients and simulated encounters for formative and summative assessments, but these have significant development and labor costs and are time consuming [14]. For practitioners after training, patient satisfaction and experience surveys are widely used, but these typically entail delayed feedback, too few items assessing communication, and insufficient specificity to support improvement. This demonstrates the need for an assessment tool that is easy to access and can produce timely, specific, and individual-level feedback.

The National Board of Medical Examiners (NBME) is an independent, not-for-profit organization whose mission is to protect the health of the public through state-of-the-art assessment of health professionals. NBME has recognized the assessment of communication skills as a priority, as evidenced by recent efforts to enhance communication assessment within the United States Medical Licensure Examination [7]. In order to support continued development of communication skills beyond the point of licensure and in recognition of needs of busy practitioners, the NBME supported the development and creation of the Video-based Communication Assessment (VCA). This paper describes the VCA-development process and the system that was ultimately created.

## Development

### Concept Development

Our goal in creating the VCA was to develop an engaging, realistic, and affordable tool that was efficient and convenient for busy clinicians. It should be flexible with regard to content and include robust feedback integrating patient’s perspective. Consistent with the NBME standards and practice, the system should result in assessments with strong psychometric qualities.

With these goals in mind, we developed a specific concept: A system that would be accessible via computer, smartphone, or tablet; would collect spoken responses; and would provide a means for gathering input from analog patients. Analog patients are naïve lay people asked to imagine themselves in the role of the patient [15,16]. We planned to gather analog patient responses using crowdsourcing—the practice of engaging people on the Web to complete a task or solve a problem. There are a variety of Web-based panels for this purpose; we utilized one of the largest and most studied—Amazon Mechanical Turk (MTurk) [17,18].

**Figure 1:**
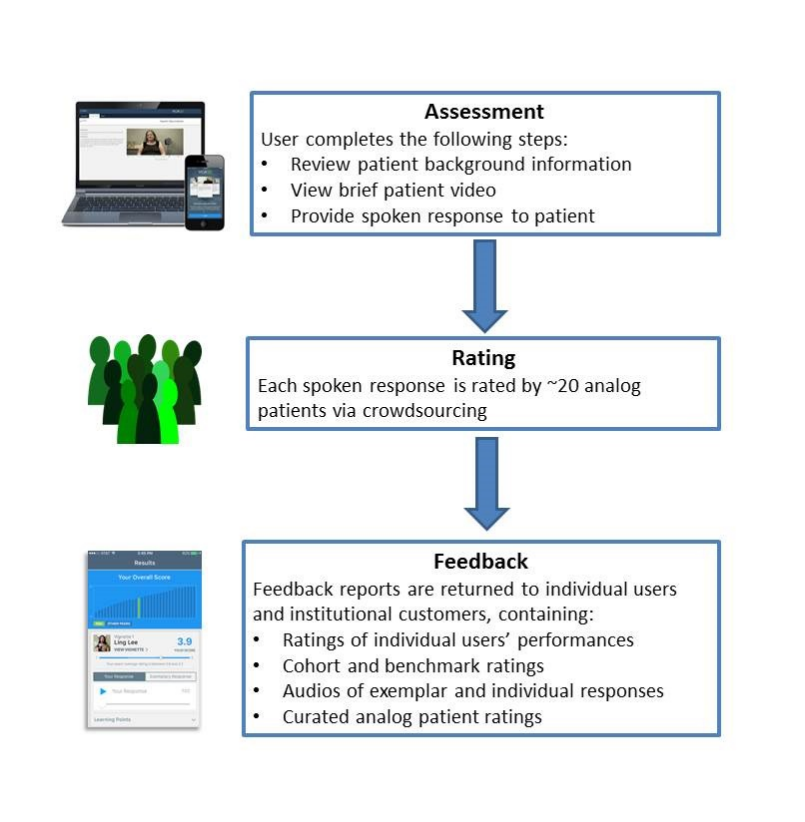
Overview of the Video-Based Communication Assessment process.

We created a prototype of the VCA that described three key elements: assessment, rating, and feedback (Figure 1). To begin, a user will log on to a dedicated website or download an app on a smartphone or tablet. The user will then be presented with brief clinical background information (in text), providing the context for a clinical encounter. The user will then click to play a brief video of the patient in the encounter. The video will end at a point where the provider will be expected to speak to the patient, and the user will be prompted to respond (ie, “What would you say next?”). The user will respond, speaking as if he or she is actually talking to the patient. This spoken response will be audiorecorded and stored. The sequence of reading a brief introduction, watching a short video, and responding as if one were talking to the patient in the encounter will be repeated for multiple vignettes. When a cohort of users completes the assessment, responses will be rated by analog patients using crowdsourcing. When ratings are completed, feedback reports will be created. These will include individual user’s ratings, comparative data on the user’s cohort; learning points derived from analysis of crowdsource raters’ comments on what will constitute a satisfactory response to the patient; and exemplary, highly rated responses that the user could compare to his or her own response.

### Concept Testing

We created a brief presentation on the VCA concept and prototype and showed it to providers, educators, and health system leaders to make an early determination of the potential attractiveness and usefulness of the VCA. Reactions were strongly positive. Encouraged by this feedback, we proceeded to develop each of the three key elements.

We then created sample vignettes, writing a brief text to provide the clinical context and producing amateur videos. We integrated these two components and the stimulus question (“What would you say next?”) into PowerPoint and, using separate recording devices, collected spoken responses from a small convenience sample of 9 clinicians. We used crowdsourcing to gather ratings of these spoken responses through a Web-based survey administered via MTurk. We demonstrated that we were able to elicit spoken responses and obtain ratings within hours of posting on MTurk.

### Creation of Video-Based Communication Assessment Content

Because the VCA is an assessment system rather than a single tool, vignette content can be created to assess diverse communication skills. For example, vignettes can be created to assess skills in providing information in a general medicine outpatient context or very specialized communication skills, such as offering an apology, delivering bad news, or describing medication side effects. We decided that the first set of VCA vignettes will be designed to assess communication skills broadly using clinical situations that would be familiar and relevant to providers from a variety of backgrounds.

**Figure 2:**
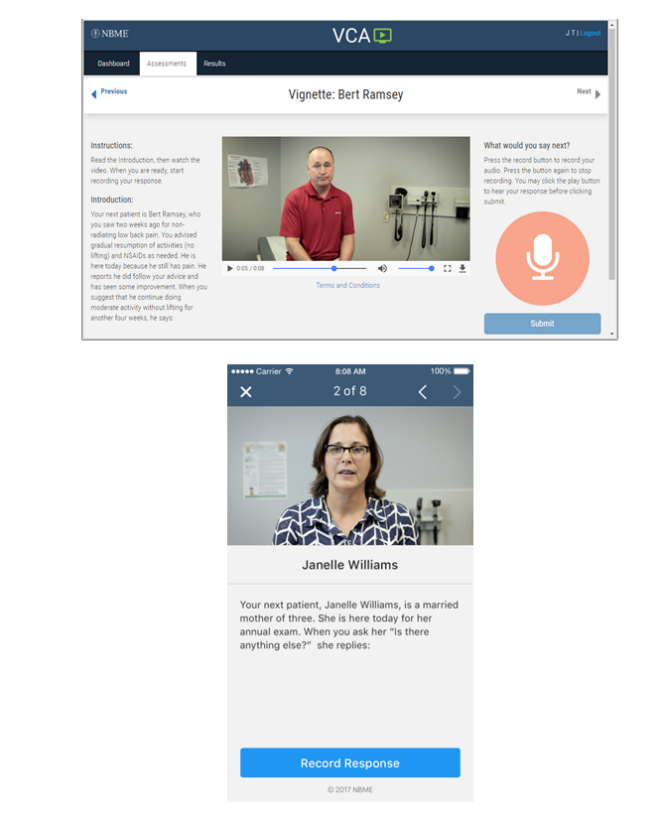
Screenshots of Video-Based Communication Assessment user interface via computer and app. (Source: Content created by National Board of Medical Examiners.).

To develop vignettes, we engaged a multidisciplinary panel of clinicians and educators to participate in a 1-day vignette-development workshop. The authors used an iterative process to refine the vignettes developed or suggested during the workshop and to generate additional vignettes. The resulting vignettes were reviewed by the authors, and corresponding videos were professionally produced. The video portrayals were assessed for realism and whether the produced vignette would be likely to appropriately stimulate a spoken response that could, in turn, be rated by analog patients. Screenshots of vignettes are presented in Figure 2.

### Development and Operationalization of the Rating Process

Consistent with our intent to incorporate the patient’s voice into the assessment, the VCA was designed to allow crowdsourced analog patients to rate users’ spoken responses. As noted above, analog patients are lay people who take on the patient’s perspective and rate the encounter as if they were the actual patient. A growing body of evidence suggests that analog patients’ ratings are reliable and valid [16] and possibly even *more* informative than actual patients’ ratings, as they avoid ceiling effects [15].

The VCA was designed to provide a seamless interface for recruiting analog patients using MTurk. MTurk is a widely used Web-based workplace that enables requesters to utilize crowdsourcing to complete specific tasks [18]. There is a growing body of literature describing demographic characteristics of MTurk workers, the quality of data collected via MTurk, and methods for improving data quality [19]. Requesters can constrain which workers may complete a task by specifying eligibility criteria (eg, workers who have consistently demonstrated a high degree of accuracy on prior tasks) or by assessing qualifications through screening questions. For instance, a requester might accept those who report that they have had a doctor’s appointment for a specific condition in the prior year.

Analog patients recruited for the VCA will first be oriented to the task and instructed to imagine themselves as the patient in the situation to be presented. The analog patient will then view a brief text (in lay language) describing the clinical context for one vignette, followed by the video of the patient speaking to the provider. He or she will then listen to a recorded response, rate the response on 2-6 items, and then proceed to the next recorded response and repeat the process. The number of responses that will be bundled together (ie, a Human Intelligence Task, as termed in MTurk) is expected to vary between 10 and 20, with the optimal number or range to be determined empirically as data are collected. Each provider’s spoken response will be rated by multiple analog patients; our prior work suggests that fewer than 15 analog patients will provide sufficient reliability [20], but the number of raters will also be determined empirically and with consideration of the purpose of the assessment. The number of analog patients needed may also vary depending on the specific vignettes used, the providers involved, and other factors; these influences will be explored in future research.

After rating all audio recordings in the bundle, the analog patients will be asked to respond to a single open-ended item, “What would you have wanted your provider to say if you had been in this situation?”

### Development of Rating Items

Because the VCA process is very different from typical communication-assessment processes, the items that analog patients use to rate users’ responses are of critical importance. We referred to three sources while developing draft rating items: (1) the 6-function model that provides the framework for communication assessment used by the NBME [7]; (2) the rating items that were used in an earlier communication assessment, which closely resembles the current VCA [20]; and (3) the Consumer Assessment of Healthcare Providers and Systems (CAHPS) item sets, which are an increasingly important point of reference for many providers and health care organizations [12]. A set of 6 items was created for pilot testing. Items will undergo extensive testing and psychometric analyses. An example of an item created for testing is “The provider explained things in a way I could understand.”

Although we anticipate that analog patients using rating items developed and tested for the VCA will be the primary way that responses are scored, the VCA system is designed to accommodate other raters and rating items. For example, researchers have expressed an interest in using the VCA to efficiently collect samples of clinicians’ typical ways of communicating with specific types of patients. Such responses could then be accessed and scored according to criteria specific to the research question. In this case, raters could be analog patients, trained research staff, or specialists selected for their expertise in the content area.

### Development of Feedback Reports

Because we anticipate that the major application of the VCA will be formative assessment, feedback is of fundamental importance. Two types of feedback reports will be created: individual and organizational. Individual feedback reports will provide detailed rating results for a single user, with aggregate, deidentified cohort results provided for comparison. To support learning, individual feedback reports will allow the users to review the vignette, replay his or her response, listen to a highly rated response, and review learning points. Learning points will be based on a content analysis of analog patients’ comments and included illustrative quotes and recommendations.

An individual user will be able to share links to his or her personal feedback report at his or her discretion. For instance, a user could choose to share a link with a trained communication coach who will work directly with him or her in one-on-one sessions discussing the feedback report, reviewing his or her responses, and comparing these to exemplary responses and comments.

Organizational level feedback reports will include summary results for an entire cohort. They will include all the components of the individual feedback reports, such as ratings, spoken responses, exemplars, and analog patient comments. In addition, these reports will allow supervisors to review the relative standing of individuals in a cohort. In the future, as the database of responses grows, multiple benchmarks will be available, showing, for instance, the average overall performance of Internal Medicine residents or Family Medicine physicians who have completed the VCA. At present, organizational level feedback reports do not contain the names of the individual users; however, this information could be included in future versions with the consent of users.

### Future Research

We have identified a number of research questions related to both operational and psychometric considerations of the VCA (Table 1). Data collection related to research questions 1-4 is currently underway. The answers to these questions will inform the final design and implementation. However, because the VCA is a system rather than a single assessment form, most of the research questions will have conditional rather than absolute answers.

**Table 1:** Priority research questions and corresponding research strategies.

Question number	Research question	Research strategy
1	Does the VCA^a^ (including the user interface, assessment process, and feedback reports) meet the needs of users and customers?	Brief postassessment surveys, user and customer interviews, market research
2	How many vignette responses, rating items, and raters will be needed to obtain a generalizability coefficient (g) of .80 or higher?	Generalizability studies
3	How does the wording of the items presented to analog patients affect ratings, and what items result in psychometrically sound scores?	Sequential testing of various items and response options, with independent samples of analog patients rating the same responses
4	To what extent are analog patient characteristics (eg, age, gender, race or ethnicity, education, geographic residence) associated with differences in ratings?	Statistical analysis of the impact of specific analog patient characteristics on ratings and assessment of the interaction between analog patient characteristics and vignette characteristics
5	Are scores on the VCA correlated with other measures of clinician-patient communication, patient experience, or patient satisfaction?	Correlational studies comparing users’ scores on the VCA with scores on relevant items from measures collected in routine practice (eg, CAHPS^b^ scores)
6	Does participating in the VCA contribute to improved provider performance?	Pre-post studies of VCA users scores on measures collected in routine practice

^a^VCA: Video-Based Communication Assessment.

^b^CAHPS: Consumer Assessment of Healthcare Providers and Systems.

## Discussion

The VCA is an innovative system for assessing clinician-patient communication. We anticipate that it will be useful to individuals and organizations seeking to improve clinicians’ communication skills, evaluate the effectiveness of training programs, or document proficiency in communication.

Both experienced and new clinicians can benefit from the VCA. Although current medical education incorporates communication skills into the standard curriculum, the addition is relatively recent. As such, there is a sizable cohort of long-practicing physicians who have never received any formal communication training and could benefit from this tool. Newer physicians have nearly all had exposure to communication basics; nonetheless, there is ample evidence from patients and families indicating that further skill building is needed [21-23]. This is particularly true around uniquely challenging topics such as end-of-life conversations, supporting and enhancing patient self-management of chronic conditions, and assisting patients struggling with pain management in the current context of pervasive opioid use disorder. Coupled with the widespread adoption of maintenance-of-certification mandates across medical specialties, clinicians have increased motivation and context for enhancing these skills.

In an organizational application, a hospital’s Chief of Medicine might use VCA results to identify clinicians who are most in need of remediation or the most skilled clinicians in order to engage those clinicians as peer coaches. VCA results could also be used to identify particular areas of strength or weaknesses for a given clinician; for example, a clinician might be identified as needing additional support regarding ways to disclose medical errors. Initial feedback on the VCA suggests that such inferences and decisions are relevant and important to hospital leaders, residency directors, and others in similar positions.

The VCA has important implications for the research setting, as it enables efficient, remote, and targeted assessment of clinicians’ communication skills in the context of descriptive and intervention studies. Vignette-based surveys have been widely used in research to explore how clinicians respond to variations in clinical situations and patient characteristics and how patients respond to variations in clinicians’ communication strategies [24]. Most often, the vignettes presented are text-based and the responses are collected via rating scales. Although some studies, particularly those using analog patients, have used video vignettes as stimuli, we are not aware of any studies, with the exception of our own prior work [20], that have captured spoken responses. There are important differences in spoken and written communication [25]. The VCA will allow researchers to efficiently study not only what clinicians say but also how they say it and how patients respond to these communicative acts. Rigorous research in this area could ultimately provide new insights into what constitutes good communication from the patient’s point of view as well as a more complete understanding of the extent to which patients’ perceptions of communication vary according to their personal characteristics.

There are a number of unanswered questions about the VCA and its impact. Specifically, further work is needed to determine whether the use of the VCA and the feedback reports, in particular, result in behavior change; whether any positive impact is obtained through the use of the report alone; or whether the support of a coach or teacher is required. Another uncertainty relates to the potential for the widespread adoption of the VCA. Although our preliminary conversations with potential customers and users were positive, it is not yet known whether institutions, organizations, programs, and individuals will find this tool attractive or useful.

Despite its many attractive features, the VCA has some limitations. The use of very brief vignettes of communication behaviors allows efficient assessment, but the lack of sustained interactions might also be considered a limitation. Our decision to use this approach was, in part, pragmatic: Doing so allows efficient assessment using a variety of vignettes in a relatively short amount of time. We recognize that this approach does not provide an assessment of clinicians’ ability to adjust their communication skills over the course of an interaction or to develop a relationship with a patient over time. At the same time, prior research suggests that this approach will result in valid and reliable scores [20] and that even brief samples of communication behaviors are informative and predictive of important outcomes [26]. We plan to conduct a series of studies to investigate the properties of VCA scores.

In summary, the VCA is a flexible, innovative system for assessing clinicians’ communication skills. Research is currently underway to provide insights into the reliability of VCA scores under various conditions and to examine the impact of rater characteristics. We believe that the properties of the VCA will enable this new system to make a substantial contribution to the assessment of communication skills and ultimately, to improving clinician-patient communication.

## Abbreviations

CAHPS: Consumer Assessment of Healthcare Providers and Systems
NBME: National Board of Medical Examiners
MTurk: Amazon Mechanical Turk
VCA: Video-Based Communication Assessment

## References

[1] Institute of Medicine Committee on Quality of Health Care in America (2001). Crossing the Quality Chasm: A New Health System for the 21st Century.

[2] Levinson W, Lesser C, Epstein R (2010). Developing physician communication skills for patient-centered care. Health Aff (Millwood).

[3] Street RL, Makoul G, Arora NK, Epstein RM (2009). How does communication heal? Pathways linking clinician-patient communication to health outcomes. Patient Educ Couns.

[4] King A, Hoppe R (2013). “Best practice” for patient-centered communication: a narrative review. J Grad Med Educ.

[5] (2017). ACGME Common Program Requirements.

[6] Liaison Committee on Medical Education Functions and Structure of a Medical School: Standards for Accreditation of Medical Education Programs Leading to the MD Degree.

[7] Hoppe R, King A, Mazor K, Furman G, Wick-Garcia P, Corcoran-Ponisciak H, Katsufrakis PJ (2013). Enhancement of the assessment of physician-patient communication skills in the United States Medical Licensing Examination. Acad Med.

[8] Rodriguez H, von Glahn T, Elliott M, Rogers W, Safran D (2009). The effect of performance-based financial incentives on improving patient care experiences: a statewide evaluation. J Gen Intern Med.

[9] Damberg CL, Raube K, Williams T, Shortell SM (2005). Paying for performance: implementing a statewide project in California. Qual Manag Health Care.

[10] (2007). Rewarding Provider Performance: Aligning Incentives in Medicare - Pathways to Quality Health Care Series.

[11] Giordano LA, Elliott MN, Goldstein E, Lehrman WG, Spencer PA (2010). Development, implementation, and public reporting of the HCAHPS survey. Med Care Res Rev.

[12] Centers for Medicare Medicaid Services (2010). Hospital consumer assessment of healthcare providers and systems (HCAHPS) survey, 2005-2008. Centers for Medicare Medicaid Services.

[13] Shirley ED, Sanders JO (2013). Patient satisfaction: Implications and predictors of success. J Bone Joint Surg Am.

[14] Patrício MF, Julião M, Fareleira F, Carneiro AV (2013). Is the OSCE a feasible tool to assess competencies in undergraduate medical education?. Med Teach.

[15] Blanch-Hartigan D, Hall JA, Krupat E, Irish JT (2013). Can naive viewers put themselves in the patients' shoes?: reliability and validity of the analogue patient methodology. Med Care.

[16] van Vliet LM, van der Wall E, Albada A, Spreeuwenberg P, Verheul W, Bensing J (2012). The validity of using analogue patients in practitioner-patient communication research: systematic review and meta-analysis. J Gen Intern Med.

[17] Sheehan K (2017). Crowdsourcing research: Data collection with Amazon’s Mechanical Turk. Communication Monographs.

[18] Buhrmester M, Kwang T, Gosling S (2011). Amazon's Mechanical Turk: A New Source of Inexpensive, Yet High-Quality, Data?. Perspect Psychol Sci.

[19] Chandler J, Shapiro D (2016). Conducting Clinical Research Using Crowdsourced Convenience Samples. Annu Rev Clin Psychol.

[20] Mazor KM, Haley H, Sullivan K, Quirk ME (2007). The video-based test of communication skills: description, development, and preliminary findings. Teach Learn Med.

[21] Fisher K, Ahmad S, Jackson M, Mazor K (2016). Surrogate decision makers' perspectives on preventable breakdowns in care among critically ill patients: A qualitative study. Patient Educ Couns.

[22] Fisher K, Smith K, Gallagher T, Burns L, Morales C, Mazor K (2017). We Want to Know: Eliciting Hospitalized Patients' Perspectives on Breakdowns in Care. J Hosp Med.

[23] Mazor KM, Roblin DW, Greene SM, Lemay CA, Firneno CL, Calvi J, Prouty CD, Horner K, Gallagher TH (2012). Toward patient-centered cancer care: patient perceptions of problematic events, impact, and response. J Clin Oncol.

[24] Converse L, Barrett K, Rich E, Reschovsky J (2015). Methods of Observing Variations in Physicians' Decisions: The Opportunities of Clinical Vignettes. J Gen Intern Med.

[25] Rubin D, Hafer T, Arata K (2000). Reading and listening to oral‐based versus literate‐based discourse. Communication Education.

[26] Ambady N, Laplante D, Nguyen T, Rosenthal R, Chaumeton N, Levinson W (2002). Surgeons' tone of voice: a clue to malpractice history. Surgery.

